# Characterization of three new serous epithelial ovarian cancer cell lines

**DOI:** 10.1186/1471-2407-8-152

**Published:** 2008-05-28

**Authors:** Véronique Ouellet, Magdalena Zietarska, Lise Portelance, Julie Lafontaine, Jason Madore, Marie-Line Puiffe, Suzanna L Arcand, Zhen Shen, Josée Hébert, Patricia N Tonin, Diane M Provencher, Anne-Marie Mes-Masson

**Affiliations:** 1 Centre de recherche du Centre hospitalier de l'Université de Montréal (CHUM)/Institut du cancer de Montréal, Montreal, Canada; 2 The Research Institute of McGill University Health Centre, Montreal, Canada; 3 Leukemia Cell Bank of Quebec and Division of Hematology, Maisonneuve-Rosemont Hospital, Montreal, Quebec, Canada; 4 Department of Medicine, Université de Montréal, Montreal, Quebec, Canada; 5 Department of Human Genetics, McGill University, Montreal, Canada; 6 Department of Medicine, McGill University, Montreal, Canada; 7 Department of Obstetrics and Gynecology, Division of Gynecologic Oncology, Université de Montréal, Montreal, Canada

## Abstract

**Background:**

Cell lines constitute a powerful model to study cancer, and here we describe three new epithelial ovarian cancer (EOC) cell lines derived from poorly differentiated serous solid tumors (TOV-1946, and TOV-2223G), as well as the matched ascites for one case (OV-1946).

**Methods:**

In addition to growth parameters, the cell lines were characterized for anchorage independent growth, migration and invasion potential, ability to form spheroids and xenografts in SCID mice.

**Results:**

While all cell lines were capable of anchorage independent growth, only the TOV-1946 and OV-1946 cell lines were able to form spheroid and produce tumors. Profiling of keratins, p53 and Her2 protein expression was assessed by immunohistochemistry and western blot analyses. Somatic *TP53 *mutations were found in all cell lines, with TOV-1946 and OV-1946 harboring the same mutation, and none harbored the commonly observed somatic mutations in *BRAF*, *KRAS *or germline BRCA1/2 mutations found to recur in the French Canadian population. Conventional cytogenetics and spectral karyotype (SKY) analyses revealed complex karyotypes often observed in ovarian disease.

**Conclusion:**

This is the first report of the establishment of matched EOC cell lines derived from both solid tumor and ascites of the same patient.

## Background

Epithelial ovarian cancer (EOC) is often described as the silent killer or the disease that whispers mainly due to absence of symptoms. This combined with the lack of specific/sensitive markers and/or techniques of screening leads to the diagnosis at late stages of the disease in more than 70% of patients. Unfortunately, the five year survival rate at this point of the disease is less than 30% [1]. Although EOC is not the most prevalent of cancers, it accounts for the highest number of deaths from a gynecologic malignancy.

EOC is a complex disease stratified according to histopathological and morphological criteria. The majority of EOCs are thought to arise from the ovarian surface epithelium (OSE) that is derived from the coelomic epithelium. OSE is composed of multipotent cells that can differentiate and give rise to tumors of different histopathology types [1,2]. The latter are defined by the International Federation of Gynecology and Obstetrics (FIGO) [3] and represent serous, endometrioid, mucinous, clear cell, de Brenner, mixed and undifferentiated subtypes. Serous type tumors are the most common subtype of EOC identified in more than 50% of cases. EOC tumors are graded according to the degree of differentiation of tumor cells which can vary from well (grade 1), moderately (grade 2) or poorly (grade 3) differentiated cells. Finally, EOC tumors are also classified according to the spread of the disease varying from stage I when tumors are confined to the ovaries to stage IV when distant metastases are observed.

Over the past years several laboratories, including ours [4], have established and characterized cell lines derived from EOC tumors. However, the majority of these EOC cell lines were established from patients ascites [4-29] and only few were derived from solid tumors [4,12,30-37]. Moreover, EOC cell lines have rarely been derived from chemotherapy-naive patients while others were established following viral transformation (SV40 Large T antigen) (such as NMSO cell line) [38,39] or xenograft passage in immunocompromised mice (such as the HEY, HO-8910PM, and AMOC-2 cell lines) [10,40,41]. In addition, few cell lines derived from serous EOC tumors are available even though this subtype represents the most frequently occurring histopathology subtype (such as the TOV-81D, FU-OV-1, and HOC1-7 cell lines) [4,10,11,33,35].

In this study, we describe three new serous EOC cell lines that were derived in our laboratory from either solid tumors or ascites of two chemotherapy-naïve patients. This is the first report characterizing cell lines derived from both solid tumor and ascites of the same patient. Moreover, the molecular and growth characteristics of the three cell lines present some unique features thereby providing the research community with new tools in the study of different aspects of serous EOC.

## Methods

### Sample and Patient data

Tumor samples were collected and banked following surgeries performed within the Division of Gynecologic Oncology at the Centre hospitalier de l'Université de Montréal (Hôpital Notre-Dame). The study was approved by the CHUM institutional ethics committee and written consent was obtained from patients prior to sample collection. Stage was determined at the time of surgery. Histopathology and tumor grade were assigned by a pathologist according to the International Federation of Gynecology and Obstetrics (FIGO) criteria [3].

### Establishment of the cell lines and culture conditions

All primary cultures and cell lines were cultured in OSE medium (Wisent, Qc, Canada) supplemented with 10% fetal bovine serum (FBS), 2.5 μg/ml amphotericin B and 50 μg/ml gentamicin. Cells were incubated in 5% CO_2 _and 5% O_2_.

The TOV-1946 cell line was established using the previously described scrape method on tumor tissue from patient 1946 [42,43]. Briefly, tumor tissue was gently scraped into a 100 mm petri dish containing supplemented OSE medium. TOV-1946 cells were maintained in the same petri dish for the first 40 days and medium was replaced weekly. After 40 days, 80% confluence was attained and TOV-1946 cells were divided into two petri dishes. They were then divided in a proportion of 2:3 once a week for the first 15 passages and 1:2 twice a week thereafter until passage 70. Subsequently, cells were maintained and divided in a proportion of 1:5 twice a week.

The OV-1946 cell line was established from a mass of cells from the ascites of patient 1946. The mass was macro-dissected into small pieces, which were kept in a 100 mm petri dish for 27 days at which point adherent cells reached 80% confluence. Pieces of tissue were then discarded. Cells were divided in a proportion of 2:3 every week for the first 15 passages and then 1:2 twice a week until passage 70. Cells were then maintained and divided in a proportion of 1:5 twice a week.

The TOV-2223 cell line was established from patient 2223 tumor tissue using the collagenase method. Briefly, tumor tissue was macro-dissected onto a 100 mm petri dish containing serum free OSE medium supplemented with 1000 U of collagenase (Sigma-Aldrich, ON, Canada). After 3–4 hours at 37°C, cells were resuspended into 8 ml of medium and the remaining tumor tissue pieces were discarded. The medium containing cells was then divided into four 60 mm petri dishes and 10% FBS was added. Cells were divided 1:2 once a week until passage 19 and then twice a week until passage 70. Subsequently, cells were maintained and divided in a proportion of 1:3 twice a week.

### Antibodies

For immunohistochemistry and western blot analyses, the following antibodies were used: beta actin AC-15 (ab6276 from Abcam inc. MA, USA), p53 (D0-1) (sc-126 from Santa Cruz Biotechnology, CA, USA), anti-c-ErbB2/c-Neu (OP15, Calbiochem, ON, Canada), Keratin 19 Ab-1 (Ms198-P0, Lab Vision Corp., CA, USA), Keratin 7 Ab-2 (MS-1352-P0) and Keratin 8 Ab-4 (MS-997-P0, both from NeoMarker, Medicorp, Qc, Canada).

### Immunohistochemistry

Formalin fixed paraffin embedded tumors were sectioned at 4 μm and the slides were stained using the immunoperoxidase method. Briefly, tissue sections were heated at 60°C for 30 minutes, deparaffinized in toluene and rehydrated in an ethanol gradient. Slides were submerged in boiling citrate buffer (0.01 M citric acid adjusted to pH 6.0) and microwaved for 10 min to unmask antigens. A 3% H_2_O_2 _treatment was used to eliminate endogenous peroxidase activity. The sections were blocked with a protein blocking serum-free reagent (DakoCytomation Inc., ON, Canada) and incubated with different antibodies for 60 min at room temperature.

The optimal concentration for each primary antibody was determined by serial dilutions. Tissues were incubated with either a secondary biotinylated antibody (DakoCytomation Inc., ON, Canada) or a rabbit anti-goat biotin-conjugated antibody (1:300) (sc-2774, Santa Cruz Biotechnology, CA, USA) for 20 min followed by incubation with a streptavidin-peroxidase complex (DakoCytomation Inc., On, Canada) for 20 min at room temperature. Reaction products were developed using diaminobenzidine containing 0.3% H_2_O_2 _as a peroxidase substrate. Nuclei were counterstained with hematoxylin and all sections were observed by light microscopy at 400× magnification. Substitution of the primary antibody with phosphate buffered saline served as a negative control.

### Growth rate

Growth rates were assessed as previously described [4]. On day 0, 1 × 10^5 ^cells were seeded onto 60 mm petri dishes. On day 1, 3, 5, 7, 9, 11 and 13 the cells were trypsinized, resuspended in medium and counted using a hemacytometer. Each experiment was performed in triplicate for each harvest and repeated once. Saturation density was defined as the mean maximum number of cells at confluence counted from two independent experiments performed with triplicates and the doubling time was calculated according to the slope of the linear portion of the growth curve.

### Anchorage independent growth in soft agarose and three-dimensional culture

Cell lines were assayed for their ability to grow in anchorage independent conditions by culturing 1 × 10^4 ^cells in agarose (0.33 g/100 ml OSE complete medium for the upper layer and 0.66 g/100 ml OSE complete medium for the base layer) [43]. Cells were cultured in soft agar for three weeks, colonies were photographed and these were used for counting. Two independent experiments performed in duplicate.

Cell lines were tested for their ability to form three-dimensional aggregates or spheroids as previously described [44,45]. Briefly, 4000 cells were suspended in 15 μl of OSE complete medium. The droplets of medium containing cells are then placed on the cover of non-coated plastic tissue culture plate. The cover is placed on a dish containing 10 ml of PBS to prevent dehydration of the droplets. The ability to form spheroids was assessed after four days.

### Low serum growth

Tumor cell growth in low serum conditions was assessed by plating cells in six well plates in OSE medium supplemented of 1% FBS, 2.5 μg/ml amphotericin B and 50 μg/ml gentamicin and cultured for 21 days. The medium was changed every seven days. The experiments were performed in duplicate.

### Wound-healing assay

Migration potential was evaluated using the scratch assay method as previously described [46-48]. Briefly, cells were plated onto a 12 well dish and once the cell confluence reached about 90% wounds were created using a 200 μl plastic tip. In order to evaluate cell migration into the wound, cells were methanol fixed and treated with Giemsa Stain (Sigma-Aldrich Inc., MO, USA) at 0, 8, 24 and 48 hours after creating the wound. The experiments were performed twice in triplicate.

### In vitro invasion assay

Cellular invasion was assayed by the ability of cells to invade a synthetic basement membrane (Matrigel, Becton-Dickenson, NJ, USA) using Boyden chambers. Polycarbonate membranes (8 μm pore size) of the upper compartment of transwell culture chambers were coated with 0.4 μg/ml Matrigel. Ovarian cancer cells were trypsinized and resuspended in OSE medium supplemented with 1% FBS. The cell suspension (20 × 10^3 ^cells/well) was placed in the upper compartment of the Boyden chamber, and the lower compartment was filled with OSE medium with 5% FBS. Cells were incubated at 37°C for 24 hours. Following incubation, membranes were methanol fixed and stained with Giemsa Stain (Sigma-Aldrich Inc., MO, USA). Non-invading cells were removed with a cotton swab, while invading cells on the underside of the membrane were counted using an inverted microscope. The experiments were performed in duplicate.

### In vivo growth in SCID mice

The tumorigenic potential of cell lines was assessed based on their ability to form tumors in 45 day-old female SCID mice at subcutaneous (s.c.) left gluteal or intraperitoneal (i.p.) injection sites. Each mouse was injected with 5 × 10^6 ^cells suspended in phosphate buffered saline (PBS). The animals were housed under sterile conditions in a laminar flow environment with *ad-lib *access to food and water. Tumor formation was assessed over 180 days. Animals were sacrificed before neoplastic masses reached limit points established by the Institutional Committee on Animal Protection (CIPA) according to the Canadian Council on Animal Care.

### Mutation analyses

*TP53 *mutations were detected by single-strand conformation polymorphism (SSCP) analysis of cell line DNA. Polymerase chain reaction (PCR) was used to amplify exons 5–9 of *TP53 *as previously described [49]. Mutations were detected as band shift relative to the wild-type pattern, and confirmed by sequence analysis (McGill University and Genome Quebec Innovation Centre, Montreal, Quebec, Canada). If negative by the SSCP assay, samples were sequenced for exons 2–11 (translated region), as previously described [50]. *KRAS *was investigated by sequencing genomic regions corresponding to codons 12 and 13 as previously described [51]. Microsatellite instability (MSI) was established as previously described [4].

*BRAF *exons 11 and 15 were analyzed by SSCP analysis. PCR was performed in a 12.5 μl volume containing 200 ng of genomic DNA; 1.25 μCi of [35S]dATP (Perkin-Elmer, ON, Canada); 1× PCR buffer (Invitrogen, ON, Canada); 2.5 nmol each dCTP, dGTP and dTTP; 0.3 nmol dATP; 1.5 mM MgCl2; 15 pmol of each primer [52]; and 0.5 U of Taq DNA polymerase (Invitrogen, ON, Canada). The PCR conditions were 3 min at 95°C, 35 cycles of 94°C for 30 sec, 55°C for 30 sec and 72°C for 30 sec. The reaction products were diluted 2:3 with stop buffer (90% formamide, 10 mM EDTA, 10% bromophenol blue and 10% xylene cyanol) and heated at 95°C for 10 min before loading on a 0.5× MDE (Mandel Scientific, ON, Canada) non-denaturing gel. The products were electrophoresed at 25 W at 4°C for 6 h. Gels were dried at 80°C and autoradiographed at room temperature for 2–3 days on Kodak Biomax MR film (Perkin-Elmer, ON, Canada). Mutations were detected as band shift relative to the wild-type pattern, and confirmed by sequence analysis (McGill University and Genome Quebec Innovation Centre).

The common French Canadian founder mutations 4446C>T and 2953delGTAinsC in *BRCA1 *and 8765delAG, 6085G>T and 3398delAAAAG in *BRCA2 *were investigated in DNA from patient matched peripheral blood lymphocytes as described [53,54].

### Conventional cytogenetics and Spectral Karyotyping (SKY) of the cell lines

Metaphase preparation and cytogenetic analyses with a trypsin-Giemsa banding technique of the TOV-2223, TOV-1946 and OV-1946 ovarian cancer cell lines were performed according to standard cytogenetic procedures. Clonal chromosomal abnormalities and GTG-banded karyotypes were described according to the International System for Human Cytogenetic Nomenclature [55]. Metaphase cells from the same culture passage were used for standard and spectral karyotyping of each ovarian cancer cell line. Slide pretreatment, hybridization with the SkyPaint™ human probes and detection were performed with the protocol provided by Applied Spectral Imaging (ASI) [56] with minor modifications. Spectral images were acquired with a SpectraCube^® ^system (ASI) mounted on a Zeiss Axioplan II microscope and analyzed using the SkyView version 1.6.1 software (ASI).

## Results

### Primary culture, cell line and tumor tissue phenotype

Cell lines were derived from both solid tumors (TOV-1946 and TOV-2223G) and ascites (OV-1946) of two chemotherapy naive patients. Patients 1946 and 2223 from which the cell lines were derived both presented poorly differentiated (grade 3) serous papillary cystadenocarcinoma at stage IIIC. Based on residual tumor present following surgery, both patients were considered to be suboptimaly debulked. Patient 2223 received a palliative treatment only and survived for 18 months while patient 1946 died from post-operative complications. Patient 1946 did not have any known familial history of cancer. However, two sisters of patient 2223 were diagnosed with ovarian cancer (70 and 80 years old) and numerous colon cancers were diagnosed within the family. Clinical data for both patients are summarized in Table 1.

**Table 1:** Patient clinical data

	Patient	
	
Clinical Parameter	1946	2223
Age at diagnosis	75	89
Tumor type	cystadenocarcinoma	cystadenocarcinoma
Histopathology sub-type	serous papillary	serous papillary
Tumor grade	G3	G3
Disease stage	IIIC	IIIC
Ascitis at surgery	Yes	yes
Surgical debulking	Sub-optimal	Sub-optimal
Progression	N/A	yes
Death	yes*	yes
Cause of death	gastric hemorrhage	disease progression
Follow up (months)	0.5	18
Treatment	N/A	Megace (palliative care only)

N/A = not applicable

*patient died from post-operative complication

**Figure 1:**
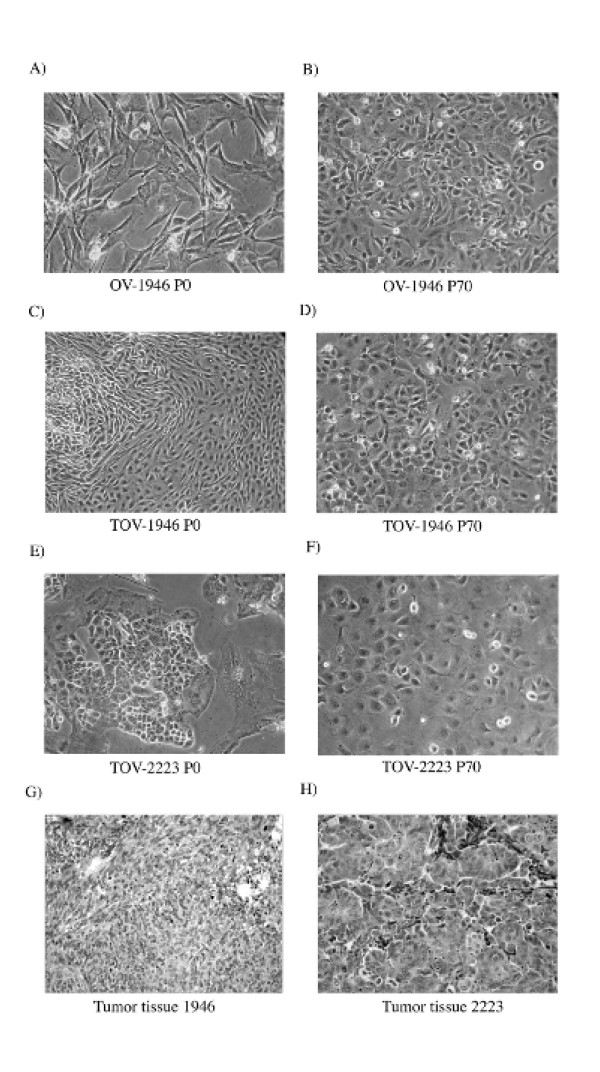
**Cellular morphology of serous ovarian epithelium cancer cell lines and their corresponding tumoral tissues**. A, C, E) Morphological appearance of the TOV-1946, OV-1946 and TOV-2223 cell lines respectively at passage 0. More (TOV-1946 and TOV-2223) or less (OV-1946) heterogenous populations of cells with obvious fibroblastic contaminants are visible for all three cell lines. B,D,F) Appearance of TOV-1946, OV-1946 and TOV-2223 cell lines respectively at passage 70. An evolution toward a more homogenous population of cobblestone-like cells typical of an epithelial cell type. G,H) Hematoxylin-Eosin stained tumor tissue sections of patient 1946 and 2223. Poorly differentiated tumor masses can be observed. All primary cultures and cell lines were cultured in OSE medium composed of 50:50 medium 199:105 (Sigma) supplemented with 10% fetal bovine serum (FBS), 2.5 μg/mL amphotericin B and 50 μg/mL gentamicin. All photographs were taken at 400× magnification.

At initial passage all three cell lines appeared more (TOV-1946 and TOV-2223) or less (OV-1946) heterogenous with populations of cells with obvious fibroblastic contaminants (Figure 1A,C,E). With subsequent passages a selection toward a more homogenous population of cells occurred and cell lines presented a cobblestone morphology characteristic of epithelial cells (Figure 1B,D,F).

Hematoxylin-Eosin stained tumor tissue sections of patient 1946 and 2223 (Figure 1G and 1H, respectively) presented typical poorly differentiated tumor masses. While all cell lines exhibited similar cobblestone morphologies typical of epithelial cells, the TOV-2223 cells are larger than the TOV-1946 and OV-1946 cells (Figure 1B,D,F). This reflects morphological differences in the corresponding tumor tissues where cells in the TOV-2223 tumors were larger than those observed in the 1946 tumor (Figure 1G,H).

One of the hallmarks of cancer cells is their ability to grow in the absence of exogenous growth factors, which can be verified by culturing cancer cells in low serum conditions. All cell lines were able to grow in a medium containing only 1% of FBS. In general however, growth rates were slower in low serum conditions than those observed in a typical 10% FBS environments (data not shown).

### Solid tumor and cell line expression of keratins, TP53 and HER2

**Figure 2:**
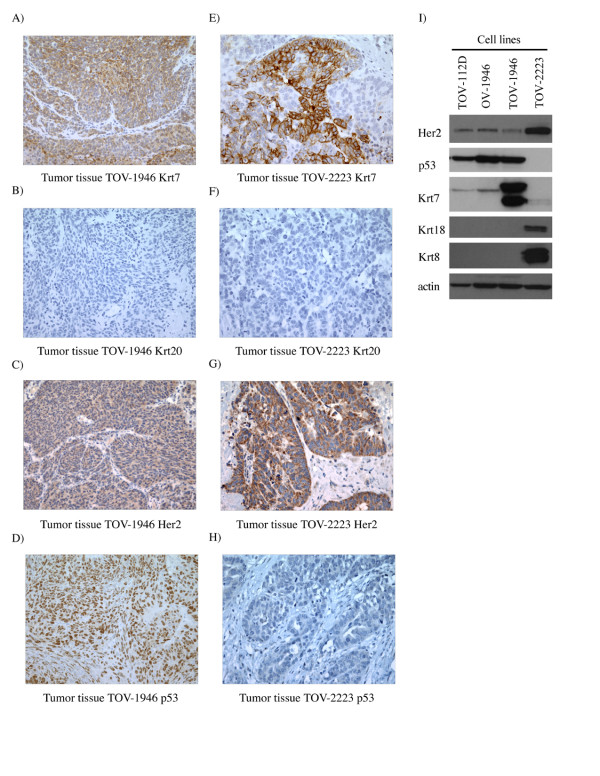
**Solid tumor and cell line expression of keratins, TP53 and HER2**. A-D) Immunohistochemistry on paraffin embedded TOV-1946 tumor tissue verifying Krt7, Krt20, Her2 and p53 expression respectively. E-H) Expression of the same four proteins was verified in the TOV-2223 tumoral tissue. Krt7 and Krt20 were used to distinguish between ovarian and gastro-intestinal tract tumors. Nuclei were counterstained with hematoxylin and all sections were observed by light microscopy at 400× magnification. I) In parallel, Her2, p53, Krt7, Krt18 and Krt8 expression was verified by western blot in cell lines. Krt7, Krt18 were used in order to confirm the epithelial character of cell lines. Actin was used as a loading control.

In order to further establish the epithelial characteristics of our cell lines keratin expression was assessed. Both TOV-1946 and OV-1946 cell lines expressed Krt7 and hence mirror keratin expression observed *in vivo *in the original tumor (Figure 2A and 2I). However, the TOV-1946 cell line expressed a much higher level of this keratin when compared to its ascites counterpart (Figure 2I). As reflected by immunohistochemistry and western-blot analyses, although TOV-2223 tumor tissue expressed Krt7, the corresponding cell line seems to have lost the capacity to express this particular keratin (Figure 2E and 2I). The epithelial characteristics of the cell lines were also verified by the expression of Krt18 and Krt8, which are also markers of epithelial cells. The expression of these keratins was observed only in the TOV-2223 cell line (Figure 2I). The gastro-intestinal tract tumor marker Krt20 was absent in both tumor tissues and all cell lines (Figure 2B and 2F).

We assessed the expression of p53 and Her2 in both original tumor tissues and cell lines. Tumor tissues from both patients exhibited Her2 expression (Figure 2C and 2G), however the level of expression was stronger in the TOV-2223 tumor tissue (Figure 2G). This is concordant with Her2 expression in the cell lines by western blot analysis (Figure 2I). We also examined *p53 *expression by immunohistochemistry. Positive staining with the *p53 *antigen is often indicative of *TP53 *gene mutation. The tissue from patient 1946 exhibited positive nuclear staining for *p53 *(Figure 2D) in contrast to that observed with the tumor from the patient 2223 (Figure 2H). *p53 *positive immunoreactivity was mirrored in the cell lines by western blot (Figure 2I).

### Cell growth rate and tumorigenicity assays

**Figure 3:**
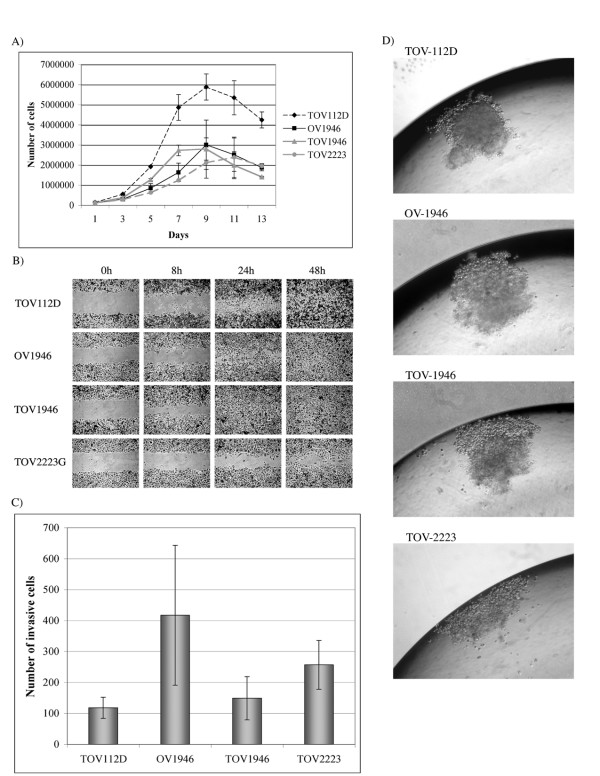
**Cell growth rate and tumorigenicity assays**. A) Growth curves of the three new cell lines as well as a previously characterized EOC TOV-112D cell line. 100 000 cells were plated onto 60 mm petri dishes. Cells were trypsinised and counted every 48 h for two weeks. Experiments were performed two times in triplicate. B) Wound-healing assay of the same four cell lines. Cells were plated onto a 12 well dish and at near confluence wounds were generated. Cells were methanol fixed and treated with Giemsa Stain at different time points in order to evaluate cell migration (0 h, 8 h, 24 h and 48 h after the scratch was performed). The experiments were performed twice in triplicate. C) Invasion assay using modified Boyden chambers. The capability of the cells to invade through matrigel membranes was verified and the invasion potential increased from TOV-112D, OV-1946, TOV-2223 to TOV-1946. The experiments were performed in duplicate. D) The capacity of the cells to form three-dimensional structures in hanging droplets was monitored. TOV-112D cells were able to form very compact spheroids, OV-1946 formed less compact spheroids, TOV-1946 cells formed loose aggregates of cells and TOV-2223 cells were unable to form any 3-D structure. Spheroid formation capability was visualized after four days.

The growth characteristics of the new cell lines were also assessed (Table 2 and Figure 3) and compared to TOV-112D, a cell line previously established and characterized in our laboratory [4]. The new EOC cell lines exhibited slower growth rates than the very aggressive TOV-112D reference cell line (Table 2 and Figure 3A). TOV-1946 has the shortest doubling time (1.3 days) when compared to the other two new cell lines (2.5 and 2.6 days for OV-1946 and TOV-2223 respectively). All cell lines exhibited similar saturation density although inferior (almost half) to that of TOV-112D. These results are consistent with the observation that TOV-112D exhibited small cells with a tendency to compact and form foci [4] as opposed to TOV-1946 and TOV-2223 cell lines which respectively show cells of medium and large size. The capacity of the new cell lines to form foci at high cell inocula and density was also verified and confirmed. The cell lines described here exhibit all the qualities of an established immortalized cell lines as they were all kept in culture for more than 150 passages.

**Table 2:** Summary of cell line growth characteristics and tumorigenicity

Cell line growth assays		TOV-1946	OV-1946	TOV-2223	TOV-112D
Growth characteristic	Doubling time (days) +/- S.D.^a^	1.3 +/- 0.4	2.5 +/- 0.9	2.6 +/- 0.7	1.0 +/- 0.2
	Saturation density (nb cells +/- S.D.)^b^	3 053 933 +/- 153 933	3 231 400 +/- 962 723	2 536 867 +/-680 852	6 162133 +/- 515 034
	Number of passages to date	>210	>210	>150	>200
Spheroid	Formation	aggregate	semi-compact	no	compact
Migration	time for wound filling (h)	24	48	>48	48
Invasion	Mean number of cells +/- S.D.^c^	417 +/- 226	149 +/- 70	257 +/- 79	118 +/- 34
Soft agarose	Colony number +/- S.D.^d^	11 +/- 3	24 +/- 8	16 +/- 4	27 +/- 10
	Colony size	large	large	small	large
Low serum	Capacity to grow in low serum (1%) conditions	yes	yes	yes	yes
Subcutaneous injection in SCID mice	Number of mice with tumors (n = 6)	0	0	0	5
	Mean time of tumor appearance (days)	N/A	N/A	N/A	7
Intraperitoneal injection in SCID mice	Number of mice with tumors (n = 6)	3	5	0	6
	Mean time of tumor appearance (days)	125	63	N/A	18
	Number of mice with ascitis	2	1	0	2

N/A not applicable

^a^calculated according to the slope of the linear portion of the growth curve.

^b^represents the mean of maximum number of cells counted among the two independent experiments performed in triplicatat

^c^number of cells that migrate through the matrigel insert

^d^mean of colony numbers per representative area (minimum 5) from two independent experiment performed in duplicate

To further characterize the new cell lines, the migration potential was assessed (Table 2 and Figure 3B). TOV-1946 cells migrated faster than the other three cell lines, as they were able to close the wound in 24 hours. The migration patterns of TOV-112D and OV-1946 were similar and filled the wound in 48 hours. However, TOV-2223 migrated more slowly and needed more than 48 hours to close the wound.

Using the modified Boyden chambers, we then measured the capability of the cells to invade through matrigel membranes (Table 2 and Figure 3C). We noted an increase in invasion potential from TOV-112D, OV-1946, TOV-2223 and TOV-1946, where TOV-112D was the least invasive and TOV-1946 the most invasive.

We next monitored the capacity of the cells to form three-dimensional structures in hanging droplets, a method routinely used in our laboratory. Only TOV-112D cells were able to form very compact spheroids, OV-1946 formed less compact spheroids, TOV-1946 cells formed loose aggregates of cells. The TOV-2223 cells were unable to form any aggregates and cells were individually spread across the droplet (Table 2 and Figure 3D).

We next measured the capability of the cell lines to grow in an anchorage independent environment by culturing the cells in soft agarose (Table 2). All cell lines were able to form colonies in soft agarose. The size of the colonies were similar for TOV-112D, TOV-1946 and OV-1946 but were larger than TOV-2223. The number of colonies increased from TOV-1946, TOV-2223, OV-1946 and TOV-112D with TOV-1946 having the lowest number of colonies and TOV112D the highest.

Finally, we monitored the potential of *in vivo *growth by injecting tumor cells at intraperitoneal or subcutaneous sites in SCID mice (Table 2). Subcutaneous tumors were observed only for the TOV-112D cell line. However, intraperitoneal (IP) tumors were also observed for the TOV1946 and OV1946 cell lines. The TOV-1946 cell line formed tumors in only 3 mice and only after an average of 125 days. The OV-1946 cell line formed tumors more rapidly (average 63 days) and a greater number of tumors (5 mice). The TOV2223 cell lines did not form tumors in mice (Table 2) however one mouse showed very small masses on liver lobes when all mice were sacrificed at approximately seven months post-injection (data not shown).

### Mutation status of the new EOC cell lines

In order to further characterize the cell lines, we performed somatic mutation analysis of *TP53*, *KRAS*, and *BRAF *as well as assayed for evidence of microsatellite instability (Table 3). Germline mutation (*BRCA1 *and *BRCA2*) analysis was limited to the recurrent mutations found in women of French-Canadian descent as cell lines were derived from patients of this ancestral origin [53]. Sequence variations in *TP53 *were identified in the DNA from all three EOC cell lines (Table 3). The same variant in exon 8, which is expected to confer an amino acid substitution of arginine to cysteine at codon 273, was identified in both TOV-1946 and OV-1946. The *TP53 *sequence variants are classified as deleterious mutations in the IARC *TP53 *mutation database [57,58]. The *TP53 *mutation status of TOV-1946 and OV-1946 is consistent with IHC results in these cell lines (Figure 2D and 2I). Although TOV-2223 harbors a *TP53 *mutation, IHC staining was negative (Figure 2). This is not inconsistent with independent reports that only a smaller fraction of cancers (about 12%) harboring nonsense mutations exhibited IHC-positivity as compared to IHC-negative nonsense mutations (IARC *TP53 *mutation database) [57]. As *TP53 *mutation analysis revealed no evidence of heterozygosity, it is likely that no functional p53 is encoded in these cell lines. No mutations in the other genes studied were found in the cell lines.

**Table 3:** Mutations status of the new EOC cell lines

Gene tested	TOV112D	TOV-1946	OV-1946	TOV-2223G
**Somatic**				

*TP53*	EX5-36 G>A, R175H	EX8+35 C>T, R273C	EX8+35 C>T, R273C	EX4+62 G>A, W53X
*KRAS*	-	-	-	-
*BRAF*	-	-	-	-
Microsatellite Instablility (MSI)	-	-	-	-

**Germline**				

*BRCA1*	-	-	-	-
*BRCA2*	-	-	-	-

- = no mutations were found

### Cytogenetics and Spectral Karyotyping

The cytogenetic alterations of the three cell lines were assessed by G-banded karyotyping and by spectral karyotyping (SKY). Twenty-five metaphases in G-banding and thirty-two metaphases in SKY (TOV-2223), twenty-two metaphases in G-banding and thirty-nine metaphases in SKY (TOV-1946) and nineteen metaphases in G-banding and fifteen metaphases in SKY (OV-1946) were analyzed. The G-banded analysis of the three cell lines revealed a modal number of 51 to 71 chromosomes with complex karyotypes that are described as a composite karyotype containing the clonal chromosomal abnormalities (Table 4) [55].

**Table 4:** G-banding composite karyotypes of the three ovarian cancer cell lines

Cell line	G-banding composite karyotypes
TOV-2223	53~71, X, der(X)t(X;2)(q13;q2?3), +der(1)t(1;17)(p3?4;q21), +add(1)(p?21), +add(1)(p12),?i(2)(q10), der(2)t(2;5)(q31;q31), +der(3)t(3;22)(q2?2;q11.2), del(3)(q23), +4, +4, -5, +5, der(6;12)(q10;q10), +add(6)(p11.2), +7, del(8)(p11.2)x2, +del(8)(p21), +9, der(10)t(5;10)(q?31;q26), +11, +add(12)(q11), +add(12)(p11.2), +add(12)(q24), +der(12;14)(p10;q10), add(13)(p11.2), +i(13)(q10), add(14)(p11.2), -15, -15, der(15)add(15)(p11.2)add(15)(q26), add(16)(q22), +add(16)(q22), -17, -17, -18, add(18)(p11.2), del(19)(p13), add(19)(q13), -20, add(21)(p11.2), -22, i(22)(q10), +20mar, inc [cp25]

TOV-1946	51~62, X, -X, der(X;12)(q10;q10), del(1)(q41q42), +add(1)(p13), +del(1)(q12), add(2)(q37), t(3;6)(p21;p21), t(3;6)(p21;p21)x2, del(3)(p?14), add(4)(q35), del(5)(p12), +der(5;8)(q10;p10), der(6)t(3;6)(p10;q10)del(6)(q?21), +der(6)add(6)(p25)add(6)(q27), del(7)(q11.2q21), +add(7)(p11.2), add(8)(p11.2), +der(8)?t(8;12)(p12;q13), +9, +del(9)(p21), +der(9)del(9) (p13)add(9)(q32), +10, add(11)(p15)x2, +del(11)(p11.2), del(12)(p11.2)x2, der(13)?t(11;13)(p11.2;p11.2)x2, add(15)(p11.2)x2, + der(15)?t(8;15)(q21;p11.2), -16, add(16)(p13), add(16)(q24), -17, add(17)(p11.2), add(17)(p1?3), add(18)(q23), +19, +add(19)(q13.4), +20, +20, -21, -22,+22,+?22,+20mar,inc[cp22]

OV-1946	57~65, X, -X, der(X;12)(q10;q10), del(1)(q41q42), add(1)(p13), +add(1)(p13), +del(1)(q12), -2, der(2)del(2)(p?14)add(2)(q?31), t(3;6)(p21;p21), del(3)(p?14), +5, +del(5)(q11.2), +7, +add(7)(q11.2), +8, +add(8)(p11.2), +der(8)?t(8;12)(p12;q13), del(9)(p21), add(9)(q32), +add(9)(q32), +der(9)del(9)(p13)add(9)(q32), +10, +add(10)(q26), add(11)(p15)x2, +del(11)(p11.2), del(12)(p11.2), der(13)?t(11;13)(p11.2;p11.2)x2, add(15)(p11.2)x2, +der(15)?t(8;15)(q21;p11.2), add(16)(p13), -17, -17, add(17)(p11.2), add(17)(p1?3), +del(17)(p11.2), -18, +18, +19, +19, +20, +20, -21, +22, +?22, +26mar, inc [cp19]

**Figure 4:**
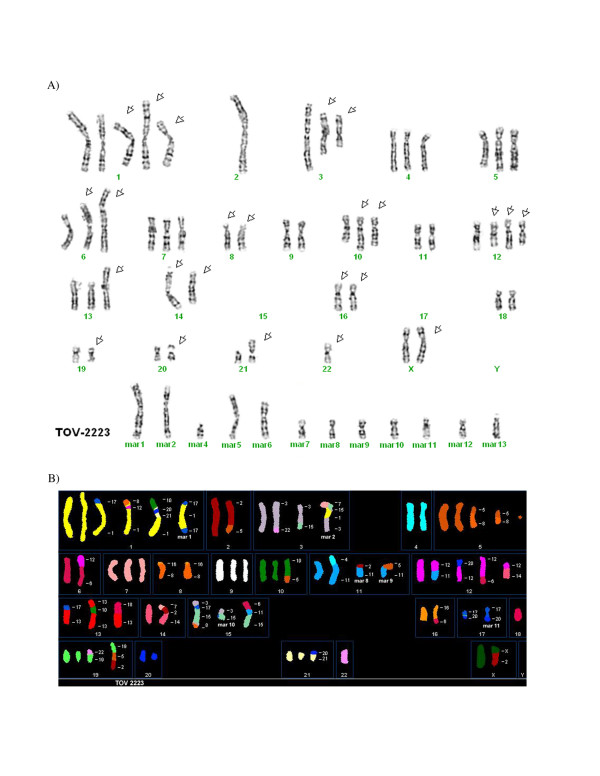
**A) Representative G-banded metaphases from the TOV-2223 (cell 10) cell line.** Arrows indicate the abnormal chromosomes, mar: marker chromosome. B) Representative metaphases from TOV-2223 (cell 45) analyzed by SKY. The origin of several marker chromosomes (mar) is defined by SKY analysis. Other examples of G-banded metaphases and the combined inverted-DAPI and SKY images of different cells are presented (see additional Files 1 and 2).

**Figure 5:**
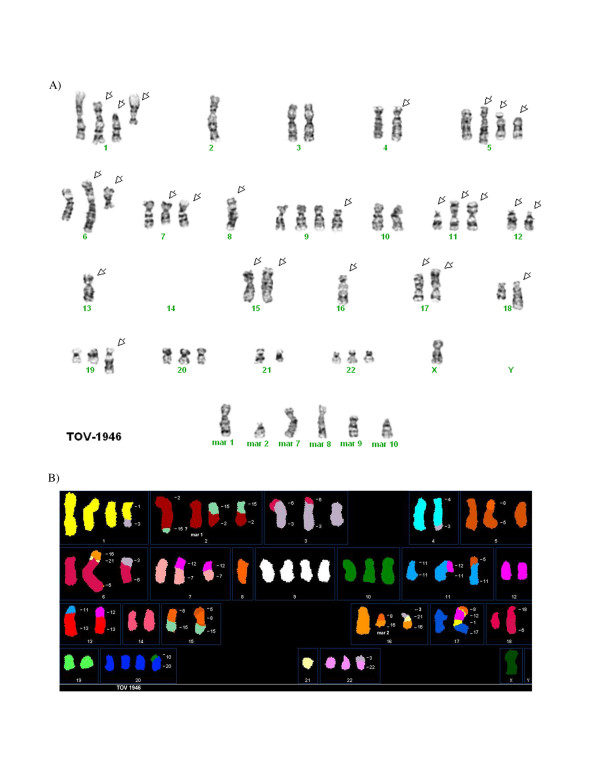
**A) Representative G-banded metaphases from the TOV-1946 (cell 34) cell lines.** Arrows indicate the abnormal chromosomes, mar: marker chromosome. B), D), F) Representative metaphases from TOV-1946 (cell 44) cell line analyzed by SKY. The origin of several marker chromosomes (mar) is defined by SKY analysis. Other examples of G-banded metaphases and the combined inverted-DAPI and SKY images of different cells are presented (see additional Files 3 and 4).

**Figure 6:**
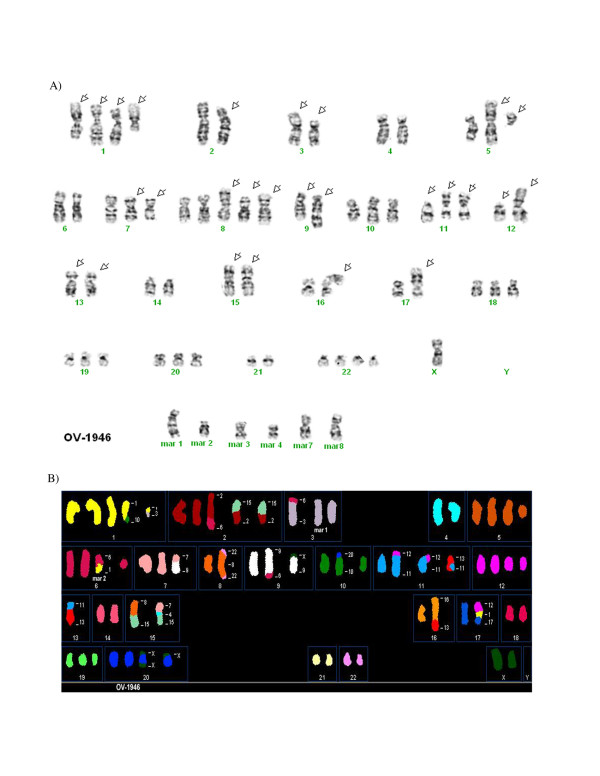
**A) Representative G-banded metaphases from the OV-1946 (cell 1) cell line.** Arrows indicate the abnormal chromosomes, mar: marker chromosome. B) Representative metaphases from OV-1946 (cell 11) cell line analyzed by SKY. The origin of several marker chromosomes (mar) is defined by SKY analysis. Other examples of G-banded metaphases and the combined inverted-DAPI and SKY images of different cells are presented (see additional Files 5).

For the definition of the chromosomal breakpoints and the characterization of the marker chromosomes, the inverted-DAPI banding and spectral images were compared with the SKY-painted chromosomes of the same cell and then studied with the G-banded karyotypes for each cell line. This method allows a better identification of the numerous chromosomal rearrangements and several markers in each cell line. It is noteworthy that there were common but also unique chromosomal abnormalities when comparing the TOV-1946 and OV-1946 cell lines. Some representative metaphases are shown in Figures 4, 5 and 6 (see also Additional Files 1, 2, 3, 4, 5).

## Discussion

In order to establish new cellular models of EOC, all samples of ovarian tissues collected for our tumor banks are processed to derive primary cell cultures. Occasionally, some of the primary cultures evolve to become immortal cell lines. So far, we have been successful in establishing seven EOC cell lines [4], three of which are described here. All cell lines were derived from patients who were never exposed to chemotherapy. This is of importance, since the majority of available cellular models originate from samples obtained following neoadjuvant therapy that could introduce genetic events not related to the biology of the disease.

Early in culture, cell lines presented heterogenous populations of cells that eventually progressed toward a more uniform population of cells with cobblestone-like morphology typical of epithelial cells. All cell lines grew as adherent monolayers without evidence of cellular piling. However, we noted that TOV-2223 cells were more adherent to the petri dish when compared to TOV- and OV-1946. Indeed, a longer trypsinization time is required to detach these cells from the petri dish. Also, when fixed in methanol, a large portion of both 1946 cell lines detached from the solid support while 2223 cells did not (data not shown). The homogeneity of cells observed at passage 0 of OV-1946 could reflect the fact that these cells came from a mass in the patient's ascites that is more homogenous then a tumor mass often composed of, among others, epithelial tumor cells, stromal cells and endothelial cells.

Cell lines were also evaluated on their capacity to survive and grow in low serum conditions. It is worth noticing that although in normal growth conditions both TOV and OV-1946 cell lines present similar growth rates, in low serum conditions the later is almost two times slower. This could be in line with the fact that the OV-1946 cell line comes from ascites which is rich in growth factors and in culture can replace FBS [59]. This cell line could hence be more dependent on growth signals provided by the environment as opposed to cells originating from solid tumors where cells may be predicted to be more self-sufficient.

In epithelial cells, intermediate filaments are composed of keratins that vary according to the differentiation of the cells. Both OSE and EOC tumors are characterized by the expression of different keratins such as *KRT7*, *KRT8 *and *KRT18*. In order to determine if our cell lines presented these EOC markers, we monitored protein expression of Krt7, Krt8 and Krt18 by western blot. When antibodies were appropriate for immunohistochemistry on paraffin embedded tissues, we also compared keratins expression in the original tumor tissues. It is also well described that monitoring expression of Krt7 and Krt20 could distinguish between ovarian and gastro-intestinal tract tumors (reviewed in [60]). Both the 1946 and the 2223 patients presented different epithelium specific Krt expression (Figure 2A and 2E) but did not express Krt20. It is worth noting that the Krt patterns were not all the same between different cell lines. Stronger expression of Krt7 is observed in TOV-1946 and TOV-2223G was the only cell line that expressed Krt18 and Krt8. This underlines the fact that these cell lines are biologically different even though they originate from tissues representing the same type of serous EOC disease. Moreover, Krt7 is also differentially expressed between the TOV-1946 and OV-1946 cell lines that not only originate from the same type of disease but also from the same patient and differences are related to solid tumor versus ascites.

In EOC, 25 to 30% of the tumors present an amplification of the *HER2 *gene leading to the overexpression of the protein Her2 (reviewed in [2,61-63]). The overexpression of this growth factor receptor alone was shown to be sufficient to induce malignant transformation and is implicated in ovarian cancer as well as many other types of cancer [64-67]. In EOCs of advanced stage, over 50% of the tumors were shown to be mutated in the *TP53 *gene (reviewed in [2,61-63]). All three cell lines showed Her2 protein expression and contained TP53 gene mutations.

Differences in growth rates, migration, invasion and spheroid formation between the TOV-1946 and TOV-2223 underline the diversity of phenotypes that can be observed within the same type of serous EOC disease. The results from the comparison of the TOV-1946 and OV-1946 growth characteristics, demonstrate that these cell lines present with unique phenotypes. Indeed, TOV-1946 cells had a better capability to invade and migrate although their capacity to form spheroid is reduced compared to the OV-1946 cells. One might speculate that cells derived from solid tumor conserve their migration and invasion property but in ascites, these characteristics are less vital. It has been previously shown that cells present in ascites form spheres or aggregates that can adhere to different extracellular matrices as well as to normal human mesothelial cells [68] but are not always invasive. The role of spheroids in ascites of ovarian cancer patients remains undefined. This is the first report of cell lines derived both from solid tumor and ascites cells of the same patient and further studies on these cell lines may provide useful insights into the biological progression of EOCs.

As both the TOV-1946 and OV-1946 injected mice developed ascites we hypothesize that the ability for cell lines to induce ascites formation in mice is intrinsic to the given cell line and independent of their origin (solid tumor versus ascites). It has been previously shown that the *in vivo *tumorigenicity can usually be predicted by the ability of the cells to grow in soft agar [69]. The OV-1946 cell line, which was the most tumorigenic, also formed the largest and most numerous colonies when seeded in soft agar (Table 2). In previous studies we have also observed that the capacity of the cells to form compact spheroids is related to their ability to form tumors *in vivo *[70]. Consistent with this notion, here we show that the OV-1946 cell line formed the most compact spheroids among the new cell lines and formed the greatest number of tumors with the shortest latency in xenograft experiments. However, the TOV-2223 cell line does not even form an aggregate in hanging droplets, only formed small colonies in soft agar and produced no tumors in SCID mice. The tumorigenicity results for TOV-2223 are also consistent with the relative indolent disease observed in patient 2223 who survived a relatively long period post diagnosis without treatment. Indeed, we have previously isolated the TOV-81D cell lines, also from an indolent disease, which failed to form tumors in immuno-compromised mice [4] suggesting inherent qualities of the tumor that are reflected in its clinical behavior can affect this biological parameter.

The unique features of the described cell lines provide complementary models for different aspects of the disease. For example, while TOV-2223 and the TOV-1946 cell lines are both derived from solid tumors of the same type of serous EOC disease, they vary in important aspects including their aggressiveness and karyotype. They may prove useful for comparative studies to uncover molecular events that distinguish very aggressive from more indolent serous disease in ovarian cancer. Although the TOV-1946 and OV-1946 lines were derived from the same patient, they were collected from the solid tumor and the corresponding ascites respectively. Although common genome modification are shared between these two cell lines (see examples of modifications occurring on chromosome X, 1, 2, 7, 8, 9, 11, 12, 13, 15 and 16) (Table 4), others are unique either to the solid tumor or the ascites derived cell line (chromosome 2, 4, 5, 6, 7, 9, 16, 17, 18, 19, 20 and 22) (Table 4). These observations suggest that the two cell lines acquired common modifications during the earlier steps of tumorigenesis and during cancer progression different rearrangements were selected for, depending on the microenvironment.

The results obtained by SKY and G-banding assays reflect previously published karyotype studies on EOC where high genomic instability is observed [71]. Recently, it as been shown that recurrent rearrangements resulting in the formation of new fusion genes could be identified using genomic and bioinformatic tools [72-75]. So far such studies have been difficult to conduct on solid tumors and hence the importance of appropriate cellular models representing different types of tumors. The future fine cartography of recurrent lesions in these cell lines may provide insights into the molecular events that contribute to EOC initiation and progression.

## Conclusion

In conclusion, this paper describes the establishment and characterization of three new serous EOC cell lines derived from two chemotherapy naïve patients. These cell lines are new and important tools in the study ovarian cancer disease, as few cell lines are described to date that represent this frequently diagnosed histopathology type of EOC. The rich characterization of these cell lines, including epithelial marker expression, growth characteristics, mutation and SKY analysis, provide the foundation for future experiments using these new models of EOC.

## Competing interests

The authors declare that they have no competing interests.

## Authors' contributions

VO and MZ performed the growth assays (growth rate, anchorage independent growth in soft agarose, three-dimensional culture and growth in low serum), the wound-healing assays, mice assays as well as writing and editing of the manuscript according to all authors revisions. LP derived and established the new cell lines from tumor samples and performed the immunohistochemistry on the primary cultures and cell lines. JM performed the immunohistochemistry on tumor tissue. JL performed the Western blot.

M–LP performed the invasion assays. SLA and ZS performed mutations analyses. JH analyzed the SKY and G-banding assays. PNT, DMP and A–MM–M contributed to the conception and design of the study as well as analysis and interpretation of the data. All authors revised the manuscript and gave final verbal approval.

## Pre-publication history

The pre-publication history for this paper can be accessed here:



## Supplementary Material

Additional file 1 A) G-banded metaphases from the TOV-2223 cell line (cells 15 and 36 respectively). Arrows indicate the abnormal chromosomes, mar: marker chromosome. B) Combined inverted-DAPI and SKY image of cell 45 and 46 respectively from the TOV 2223 cell line with identification of some marker chromosomes. Click here for file

Additional file 2 A) G-banded metaphases from the TOV-2223 cell line (cells 15 and 36 respectively). Arrows indicate the abnormal chromosomes, mar: marker chromosome. B) Combined inverted-DAPI and SKY image of cell 45 and 46 respectively from the TOV 2223 cell line with identification of some marker chromosomes. Click here for file

Additional file 3 A) G-banded metaphases from the TOV-1946 cell line (cells 6 and 43 respectively). Arrows indicate the abnormal chromosomes, mar: marker chromosome. B) Combined inverted-DAPI and SKY image of cell 44 and 36 respectively from the TOV-1946 cell line with identification of some marker chromosomes. Click here for file

Additional file 4 A) G-banded metaphases from the TOV-1946 cell line (cells 6 and 43 respectively). Arrows indicate the abnormal chromosomes, mar: marker chromosome. B) Combined inverted-DAPI and SKY image of cell 44 and 36 respectively from the TOV-1946 cell line with identification of some marker chromosomes. Click here for file

Additional file 5 A) G-banded metaphase from the OV-1946 cell line (cell 24). Arrows indicate the abnormal chromosomes, mar: marker chromosome. B) Combined inverted-DAPI and SKY image of cell 11 from the OV-1946 cell line with identification of some marker chromosomes. Click here for file
