# Supplementary figures and images for: Characterization of three new serous epithelial ovarian cancer cell lines
Source: BMC Cancer. 2008 May 28;8:152. doi: 10.1186/1471-2407-8-152 (PMC2467432; doi:10.1186/1471-2407-8-152)

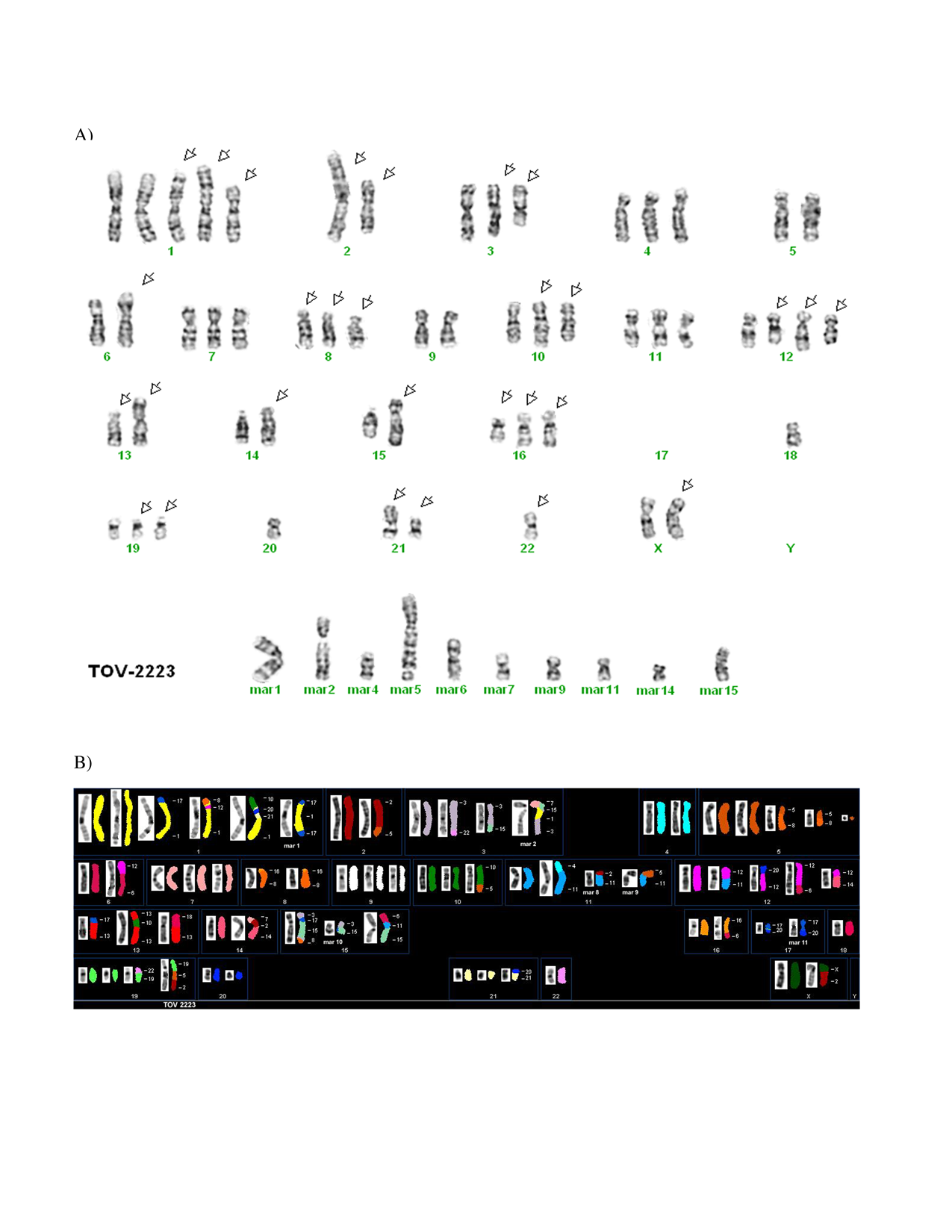

Supplement: Additional file 1 — A) G-banded metaphases from the TOV-2223 cell line (cells 15 and 36 respectively). Arrows indicate the abnormal chromosomes, mar: marker chromosome. B) Combined inverted-DAPI and SKY image of cell 45 and 46 respectively from the TOV 2223 cell line with identification of some marker chromosomes. [file 1471-2407-8-152-S1.tiff]

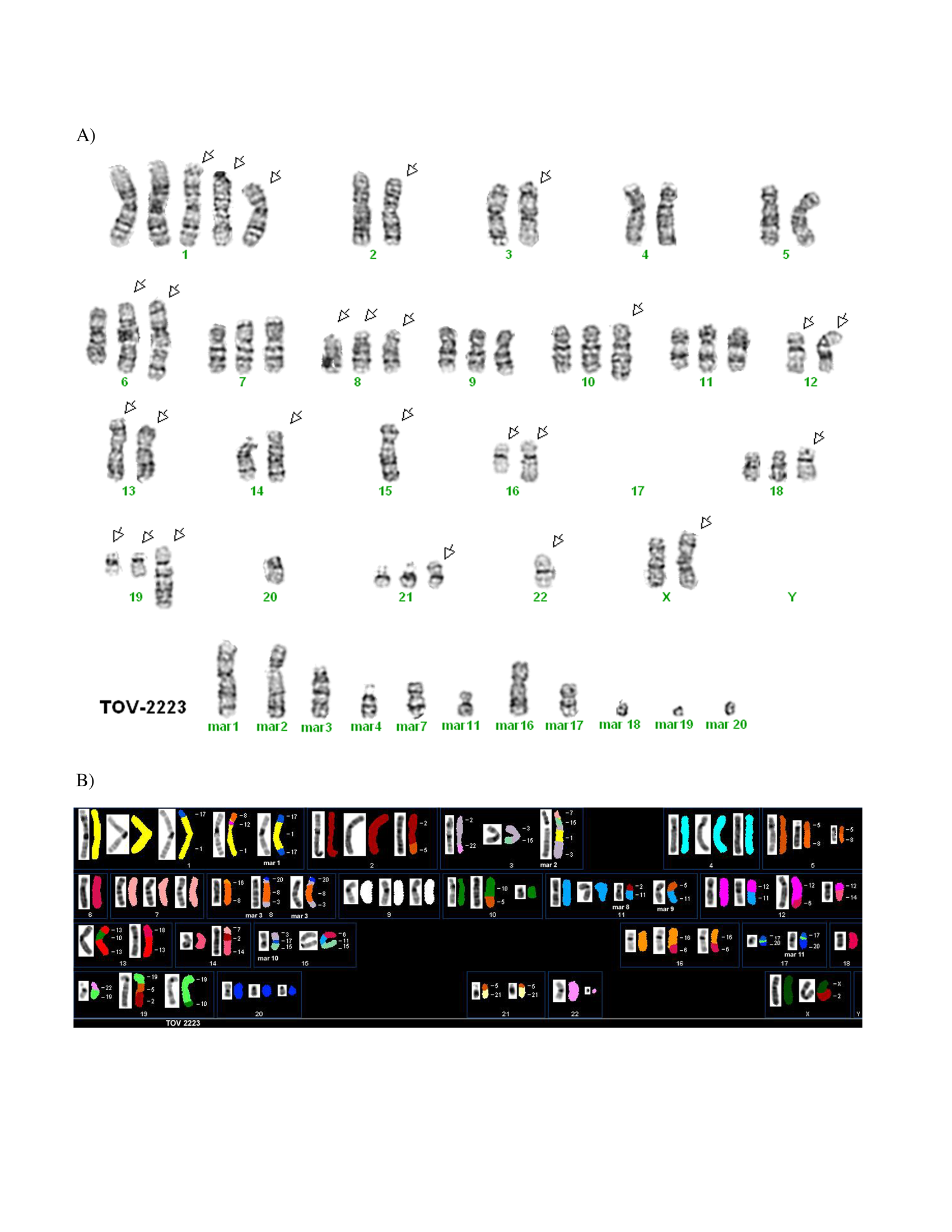

Supplement: Additional file 2 — A) G-banded metaphases from the TOV-2223 cell line (cells 15 and 36 respectively). Arrows indicate the abnormal chromosomes, mar: marker chromosome. B) Combined inverted-DAPI and SKY image of cell 45 and 46 respectively from the TOV 2223 cell line with identification of some marker chromosomes. [file 1471-2407-8-152-S2.tiff]

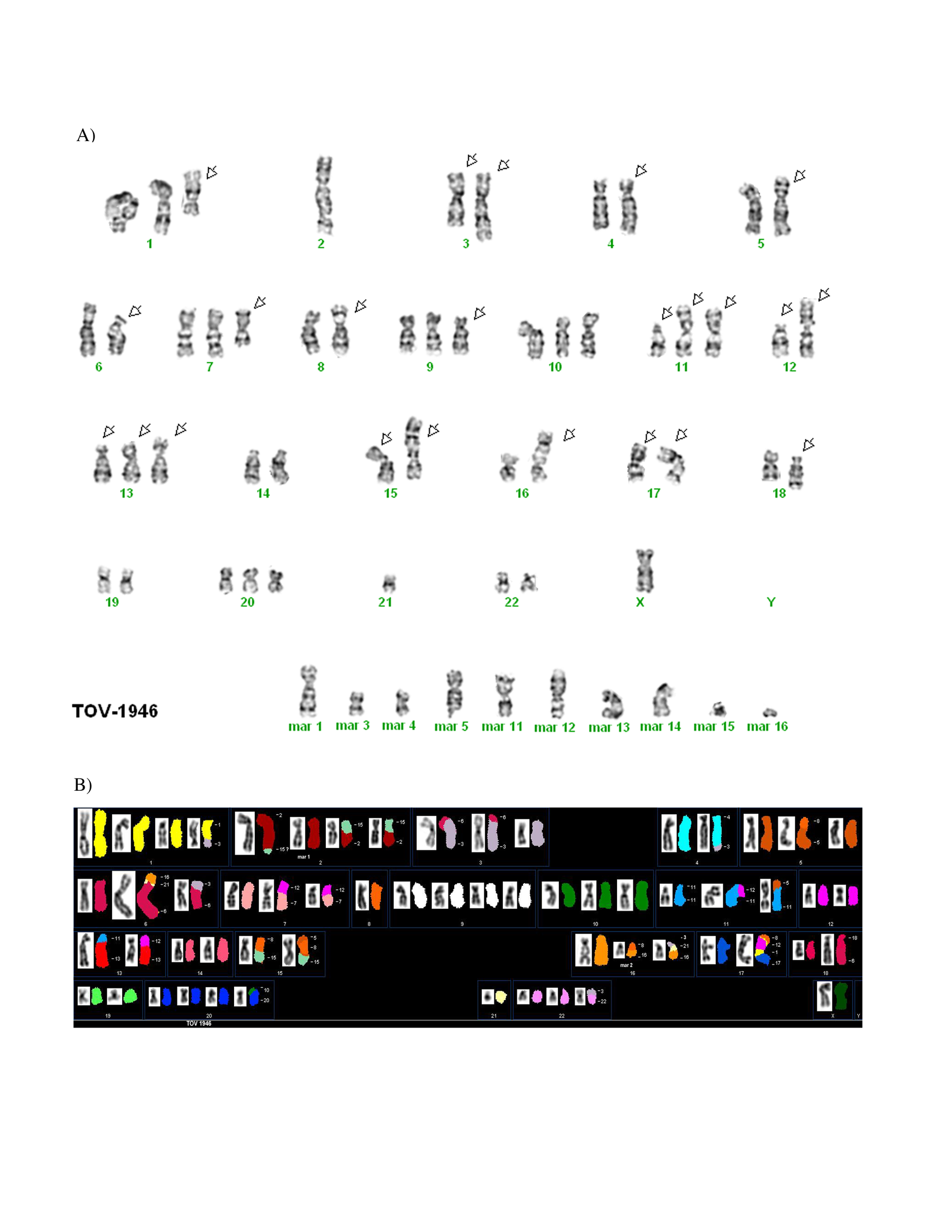

Supplement: Additional file 3 — A) G-banded metaphases from the TOV-1946 cell line (cells 6 and 43 respectively). Arrows indicate the abnormal chromosomes, mar: marker chromosome. B) Combined inverted-DAPI and SKY image of cell 44 and 36 respectively from the TOV-1946 cell line with identification of some marker chromosomes. [file 1471-2407-8-152-S3.tiff]

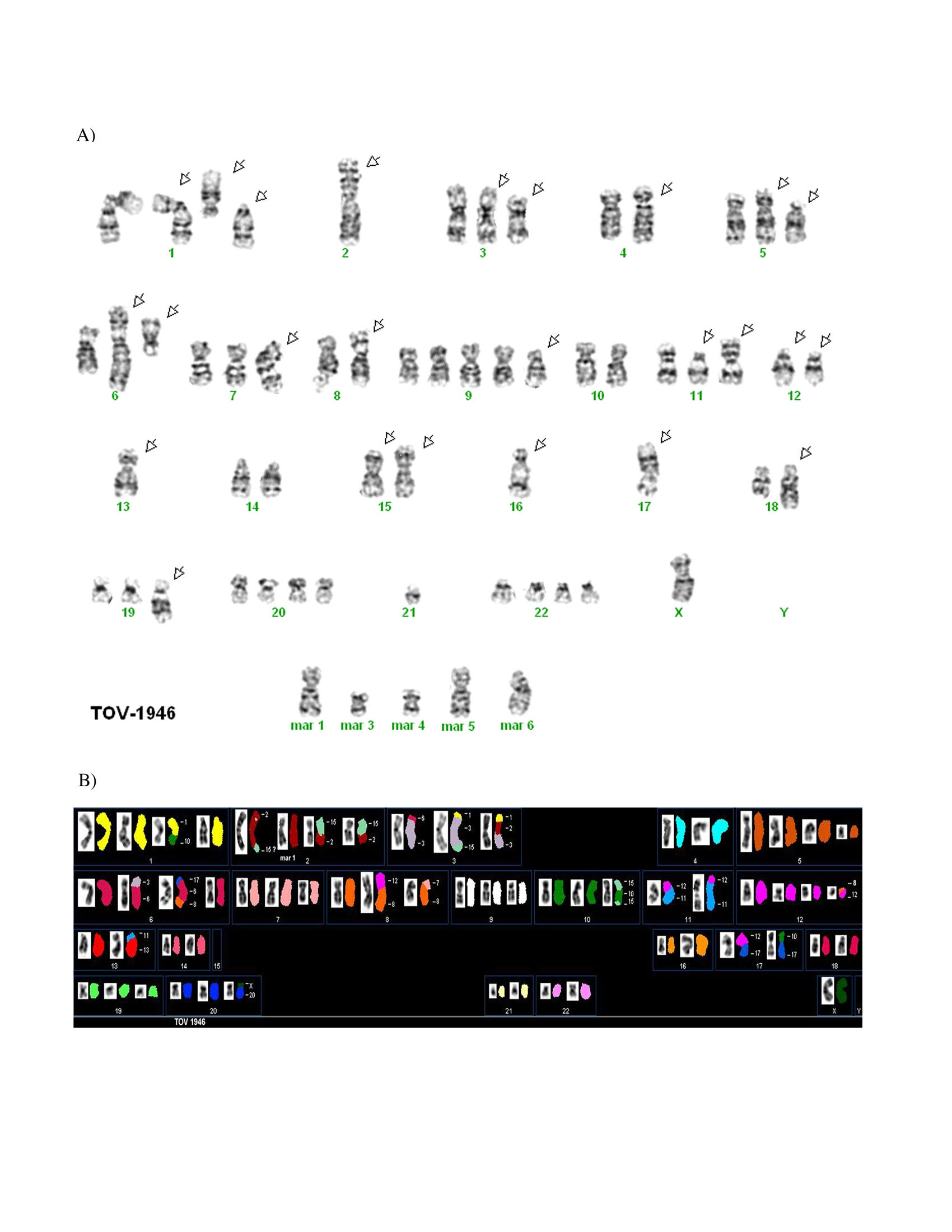

Supplement: Additional file 4 — A) G-banded metaphases from the TOV-1946 cell line (cells 6 and 43 respectively). Arrows indicate the abnormal chromosomes, mar: marker chromosome. B) Combined inverted-DAPI and SKY image of cell 44 and 36 respectively from the TOV-1946 cell line with identification of some marker chromosomes. [file 1471-2407-8-152-S4.tiff]

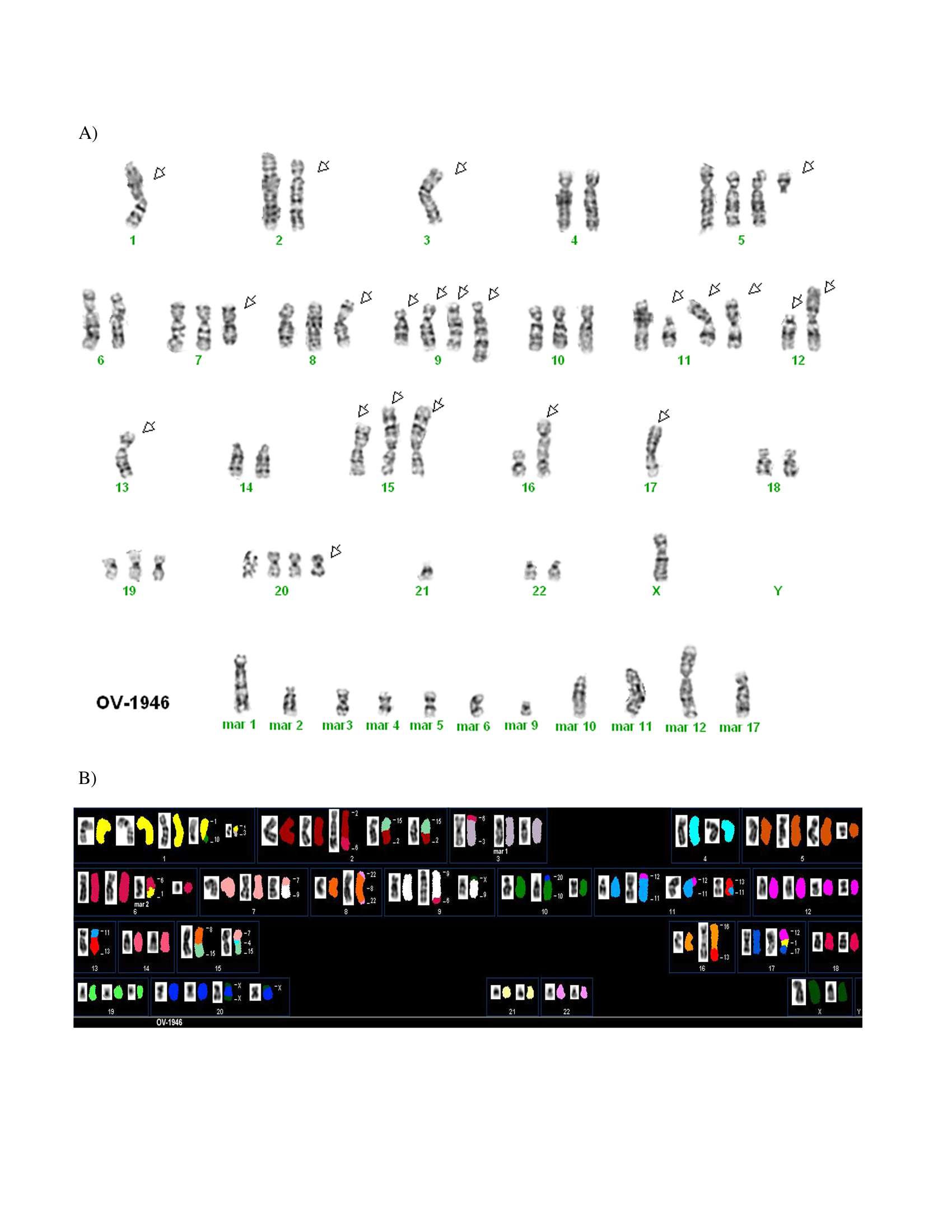

Supplement: Additional file 5 — A) G-banded metaphase from the OV-1946 cell line (cell 24). Arrows indicate the abnormal chromosomes, mar: marker chromosome. B) Combined inverted-DAPI and SKY image of cell 11 from the OV-1946 cell line with identification of some marker chromosomes. [file 1471-2407-8-152-S5.tiff]
